# Preparation and Characterization of Rodent Intestinal Microsomes: Comparative Assessment of Two Methods

**DOI:** 10.4103/0250-474X.51968

**Published:** 2009

**Authors:** Anagha Damre, S. R. Mallurwar, D. Behera

**Affiliations:** Drug Metabolism and Pharmacokinetics, Piramal Life Sciences Limited, Goregaon (E), Mumbai-400 063, India; 1 School of Pharmacy and Technology Management, NMIMS University, Vile-Parle (W), Mumbai- 400 056, India

**Keywords:** Intestinal microsomes, CYP450, Drug metabolism, Rodent

## Abstract

Small intestine plays an important role in the first-pass metabolism of orally ingested xenobiotics as a result of expression of both Phase I and Phase II metabolic enzymes, together with associated transporters. Intestinal microsomes thus can be used to study susceptibility of compounds to metabolism *in vitro*. The present study was undertaken to have a comparative assessment between different methods of preparation of rodent intestinal microsomes. Mouse and rat intestinal microsomes were prepared by two methods, in method A intestines were homogenized, while in method B mucosal cells were scrapped followed by homogenization. Further, microsomes were prepared by centrifugation (10000xg) followed by ultra centrifugation (100000×g) of the homogenates. The prepared microsomes were characterized for protein concentration using Bradford's method and CYP450 content using carbon monoxide bubbling method. The protein concentration and CYP450 content in microsomes prepared by method B was significantly higher than method A. In conclusion, superior quality intestinal microsomes can be obtained from rodents by using scrapped intestinal mucosal cells as compared to the intestinal homogenates.

Liver plays a major role in drug metabolism; however, significant first pass metabolism of xenobiotics by the intestinal wall has been increasingly recognized[1], which affects the oral bioavailability of compounds. This is supported by the expression of numerous Phase I and Phase II metabolic enzymes, to add to the positioning of small intestine as the first site of exposition of xenobiotics to metabolic system, and the large surface area available in the small intestine for absorption of xenobiotics. In addition, the small intestine is sensitive to induction and inhibition of drug metabolizing enzymes[2] added to the interplay between transporters and metabolic activities[3]. Like humans, several P450s are expressed in the rodent intestines namely CYP1A, 2B, 2C, 2D and 3A along with conjugation enzymes like glutathione-S-transferases and UDP-glucuronyl transferases. *In vitro* intestinal systems, like microsomal preparations and S9 fractions, have been widely used for studying metabolic fate of compounds. Different methods have been reported in literature for preparation of intestinal microsomes. Lu *et al*.[4] have reported preparation of intestinal microsomes from intestinal mucosal cells scrapped out of the intestinal wall. Walker *et al*.[5] have reported direct homogenization of intestinal segments for microsomal preparations. However, there is no data available to evaluate the quality of microsomes obtained following either of these methods. Thus, in the present investigation, we have undertaken comparative assessment of preparation of intestinal microsomes using these two methods with some modifications for application in drug metabolism studies.

The present study was approved by the Institutional Animal Ethics Committee (IAEC) of the Research Centre. Triton X-100, sodium dithionite, urethane, sucrose, Tris, EDTA, dithiothreitol, phenylmethylsulphonylfluoride, Bradford's reagent kit were purchased from Sigma Chemical Co., USA. All other chemicals used were of analytical grade and purchased from commercial suppliers. Briefly intestinal microsomes were prepared using the following methods; method A, in which, rats (n=4) and mice (n=8) were anaesthetized with intra-peritoneal injection of urethane (10 ml/kg). Small intestinal tissues (20-25 cm proximal from the cecum) were excised rapidly and tissues were washed in chilled isotonic saline to remove excess blood. The adherent connective tissues and fat were removed. The intestinal lumen was then flushed with isotonic saline to remove partially digested food residues. The intestines were cut into small pieces and thoroughly mixed to obtain a homogenous sample. The samples were weighed, and each tissue was homogenized into two volumes of sucrose-TKM buffer (sucrose 0.25 M, Tris 80 mM, KCl 25 mM, MgCl_2_ 5 mM, pH 7.4) on ice. The homogenates obtained were centrifuged at 10000×g for 10 min at 4° to remove gross tissues and fat. The supernatants (S9 fractions) were centrifuged at 15000×g for 15 min at 4° to remove nuclei and mitochondria. Supernatants were centrifuged at 100000×g for 60 min at 4° to pellet down microsomes. The microsomal pellets were rinsed twice with 5 ml of sucrose-TKM buffer and resuspended in a volume of same buffer (volume in ml as organ weight in g).

In the method B, rats (n=4) and mice (n=8) were anaesthetized with intra-peritoneal injection of urethane (10 ml/kg). Small intestinal tissues (20-25 cm proximal from the cecum) were excised rapidly and irrigated with ice-cold 1.14% potassium chloride solution. The lumen was cut open longitudinally and washed with potassium chloride solution. Then, the upper villus layer of the mucosa was removed with the edge of a glass slide and mucosal cells were suspended in cold homogenization buffer (100 mM potassium phosphate buffer, pH 7.4, 1 mM sodium EDTA, 150 mM KCl, 0.1 mM dithiothretiol, 250 mM sucrose with 0.25 mM phenylmethylsulfonyl fluoride in methanol). The mucosal cells were pelleted by spinning at 3000×g for 6 min at 4°. Pellets were washed twice with potassium phosphate homogenization buffer. The cell pellets were homogenized in 4-fold volume of homogenization buffer and then sonicated for 30 sec at 4°. The homogenates were centrifuged at 12000×g for 35 min at 4° to sediment nuclei and mitochondria. The supernatants (S9 fractions) were centrifuged at 100000×g for 70 min at 4° to sediment the microsomal fraction. The firmly packed pellets of microsomes were resuspended by homogenization in a volume of buffer (volume in ml as organ weight in g) containing 100 mM potassium phosphate buffer, pH 7.4 containing 250 mM sucrose and 1 mM EDTA. The prepared intestinal microsomes were stored at −70° until characterization for protein concentration and CYP450 content.

Protein concentration was determined using Bradford's method using bovine serum albumin (BSA). BSA standard (1 mg/ml) was prepared in 0.1 M potassium phosphate buffer (pH 7.4). Linearity range of standard BSA was 20 μg/ml to 500 μg/ml. For estimation of protein concentration, microsomal samples were diluted 50 and 100 times, following which 20 μl of each standard BSA sample and microsomal samples were placed in wells of a 96- well plate in triplicates. Bradford's reagent (250 μl) was added to each well. The plate was incubated for 5 min to allow development of blue colour. Absorbance was read at 595 nm using an UV spectrophotometer with 96 well plate reader (Spectramax 340 PC). The protein concentration of intestinal microsomes was extrapolated directly from the BSA standard curve of absorbance versus protein concentration.

CYP450 content was determined using CO bubbling method as described by Omura and Sato[6]. Briefly, the microsomes were diluted in 1:4 ratio with 0.1 M phosphate buffer, pH 7.4 containing 0.5% Triton X-100 and 1 mM EDTA. The solution was thoroughly mixed and divided into sample and reference cuvettes. Both the cuvettes were saturated with 30 to 40 bubbles of CO at a rate of 1 bubble/sec. Once the samples were equilibrated with carbon monoxide, the baseline spectrum was recorded from 450 to 490 nm using UV spectrophotometer (Shimadzu UV 1601). A pinch of sodium dithionite was added to the sample cuvette and spectrum was recorded from 450 to 490 nm, to obtain reduced carbon monoxide *Vs* oxidized carbon monoxide difference spectrum. Extinction coefficient of 0.106 μM^−1^ cm^−1^ was used for the calculation.

CYP450 content of intestinal microsomes was calculated using the formula, CYP450 content (nmol/ml) = ((A_450_-A_490_)/0.106) × DF, where A_450_ is the Absorbance at 450 nm, A_490_ is the absorbance at 490 nm and DF is the dilution factor.

Protein concentration and CYP450 content of the prepared rat and mouse intestinal microsomes obtained by both methods is shown in Table 1. A range of CYP450 enzymes have been identified in small intestine of rodents, which are responsible for significant metabolism of xenobiotics. The expression of metabolic enzymes varies within the small intestinal villus with the highest found in mature enterocytes lining the villus tips[7], which can be obtained by scrapping of mucosal cells. The present study showed that, microsomes prepared from the scrapped mucosal cells (method B) resulted in a better CYP450, specific CYP450 and protein content compared to microsomes prepared by homogenization (method A), indicating microsomes with a higher metabolic capacity. This could be because method B provides high exposure to CYP's present in mucosal cells of the upper villus layer of intestinal mucosa. Thus, preparation of rodent intestinal microsomes using mucosal cells of intestinal wall would be a more suitable method for obtaining superior quality of intestinal microsomes having a higher protein concentration and CYP450 content.

**TABLE 1:** PROTEIN CONCENTRATION AND CYP450 CONTENT OF RAT AND MOUSE INTESTINAL MICROSOMES

Method of Preparation	Species	CYP450 content (nmoles/ml)*	Volume of microsomes (ml)	Total CYP450 content (nmoles)*	Protein concentration (mg/ml)*	Specific CYP450 content (nmoles/mg of protein)
A	Rats	0.70±0.033	8.3	5.83±0.073	8.23±0.619	0.085
(Homogenization)	Mice	0.49±0.000	5.2	2.57±0.000	9.11±1.701	0.054
B	Rats	5.28±0.000	10.22	53.96±0.000	26.83±0.470	0.196
(Scrapping)	Mice	4.29±0.067	5.92	25.43±0.377	33.66±1.362	0.127

* All values reported in the table represent the Mean±SD. Table shows the volume of microsomes obtained (ml) and the calculated values of CYP450 content (nmoles/ ml, total CYP450 content (nmoles), protein concentration (mg/ml) and specific CYP450 content (nmoles/mg of protein) obtained for mice and rat microsomes prepared by homogenization (method A) and from scrapped mucosal cells (method B). Microsomes prepared from scrapped mucosal cells showed higher CYP450 content, total CYP450 content, protein concentration and specific CYP450 content than microsomes prepared by homogenization.

## References

[1] Von Richter O, Greiner B, Fromm MF, Fraser R, Omari T, Barclay ML (2001). Determination of *in vivo* absorption, metabolism and transport of drugs by the human intestinal wall and liver with novel perfusion technique. Clin Pharmacol Ther.

[2] Pelkonen O, Boobis AR, Gundert-Remy U (2001). *In vitro* prediction of gastrointestinal absorption and bioavailability: An experts meeting report. Eur J Clin Pharmacol.

[3] Suzuki H, Sugiyama Y (2000). Role of metabolic enzymes and efflux transporters in the absorption of drugs from the small intestine. Eur J Pharm Sci.

[4] Lu X, Li C, Fleisher D (1998). Cimetidine sulfoxidation in small intestinal microsomes. Drug Metab Dispos.

[5] Walker SA, Whitten LB, Seals GB, Lee WE, Archibong AE, Ramesh A (2006). Inter-species comparison of liver and small intestinal microsomal metabolism of fluoranthene. Food Chem Toxicol.

[6] Omura T, Sato R (1964). The carbon monoxide-binding pigment of liver microsomes I. Evidences for its hemoprotein nature. J Biol Chem.

[7] Galetin A, Houston JB (2006). Intestinal and hepatic metabolic activity of five cytochrome P450 enzymes: Impact on prediction of first-pass metabolism. J Pharmacol Exp Ther.

